# Proposal of a new genomic framework for categorization of pediatric acute myeloid leukemia associated with prognosis

**DOI:** 10.21203/rs.3.rs-2925426/v1

**Published:** 2023-05-29

**Authors:** Masayuki Umeda, Jing Ma, Tamara Westover, Yonghui Ni, Guangchun Song, Jamie L. Maciaszek, Michael Rusch, Delaram Rahbarinia, Scott Foy, Benjamin J. Huang, Michael P. Walsh, Priyadarshini Kumar, Yanling Liu, Yiping Fan, Gang Wu, Sharyn D. Baker, Xiaotu Ma, Lu Wang, Jeffrey E. Rubnitz, Stanley Pounds, Jeffery M. Klco

**Affiliations:** 1 Department of Pathology, St. Jude Children’s Research Hospital, Memphis, TN, USA.; 2 Department of Biostatistics, St. Jude Children’s Research Hospital, Memphis, TN, USA.; 3 Department of Computational Biology, St. Jude Children’s Research Hospital, Memphis, TN, USA.; 4 Department of Pediatrics, University of California San Francisco, San Francisco, CA, US; 5 Center for Applied Bioinformatics, St. Jude Children’s Research Hospital, Memphis, TN, USA.; 6 Division of Pharmaceutics and Pharmacology, College of Pharmacy, Comprehensive Cancer Center, The Ohio State University, Columbus, OH, USA.; 7 Department of Oncology, St. Jude Children’s Research Hospital, Memphis, TN, USA.; 8 These authors contributed equally

## Abstract

Recent studies on pediatric acute myeloid leukemia (pAML) have revealed pediatric-specific driver alterations, many of which are underrepresented in the current classification schemas. To comprehensively define the genomic landscape of pAML, we systematically categorized 895 pAML into 23 molecular categories that are mutually distinct from one another, including new entities such as *UBTF* or *BCL11B*, covering 91.4% of the cohort. These molecular categories were associated with unique expression profiles and mutational patterns. For instance, molecular categories characterized by specific HOXA or HOXB expression signatures showed distinct mutation patterns of RAS pathway genes, *FLT3*, or *WT1*, suggesting shared biological mechanisms. We show that molecular categories were strongly associated with clinical outcomes using two independent cohorts, leading to the establishment of a prognostic framework for pAML based on molecular categories and minimal residual disease. Together, this comprehensive diagnostic and prognostic framework forms the basis for future classification of pAML and treatment strategies.

## Introduction

Pediatric acute myeloid leukemia (pAML) is characterized by aberrant clonal expansion of hematopoietic progenitors with differentiation defects^1–4^. Although pAML shares many clinical and pathological characteristics with adult AML, genetic differences have also been appreciated^5–7^. Notably, t(11;x), resulting in *KMT2A* rearrangements, are more common in pAML, and adult AML frequently harbors mutations in *DNMT3A* and splicing factor genes, while core binding factor (CBF) AMLs are common across the age spectrum^5^. Additionally, progress in diagnostic technologies has led to the identification of cryptic fusions of *NUP98*^8^ and *GLIS* family^9^ members and complex *UBTF* tandem duplications^10^ that are enriched in pAML. Recent updates in the WHO classification^11^ (WHO^5th^) and the International Consensus Classification (ICC)^12^ define AMLs with *KMT2A* and *NUP98* rearrangements with various partners as distinct disease entities. However, a substantial number of more recently discovered recurrent driver alterations in pAML continue to be categorized as “acute myeloid leukemia with other defined genetic alterations” or “AML, not otherwise specified (NOS)”, confirming the need to understand both the biological features of pAMLs with these other driver alterations and their clinical significance.

Accumulation of clinical outcomes associated with gene alterations enabled the risk stratification of adult AML according to detailed mutational profiling. The ELN2022 risk stratification^13^ incorporated various fusions, *NPM1* mutations, *FLT3*-ITD, and myelodysplastic syndrome-related changes, including chromosomal alterations and somatic mutations. In contrast, risk stratification for pAML is still developing, and various strategies are utilized in clinical trials^14,15^. This is partly due to differences in the genetic background between adult and pediatric AML^5^, the rarity of the disease^16^, and a shortage of clinical outcome studies related to genetic alterations.

To clarify the genomic landscape of pAML and its association with clinical outcomes, we characterized 895 cases of pAML by transcriptome and genome profiling. These analyses resulted in 23 molecular categories, defined by mutually exclusive gene alterations and specific expression profiles, that show unique biological characteristics and mutational backgrounds. We further determined that these molecular categories have predictive value regarding clinical outcomes that can be leveraged to establish a framework for diagnosis and outcome prediction by investigating an independent clinical study cohort.

## Results

### Comprehensive genetic characterization of pAML

**Figure 1:**
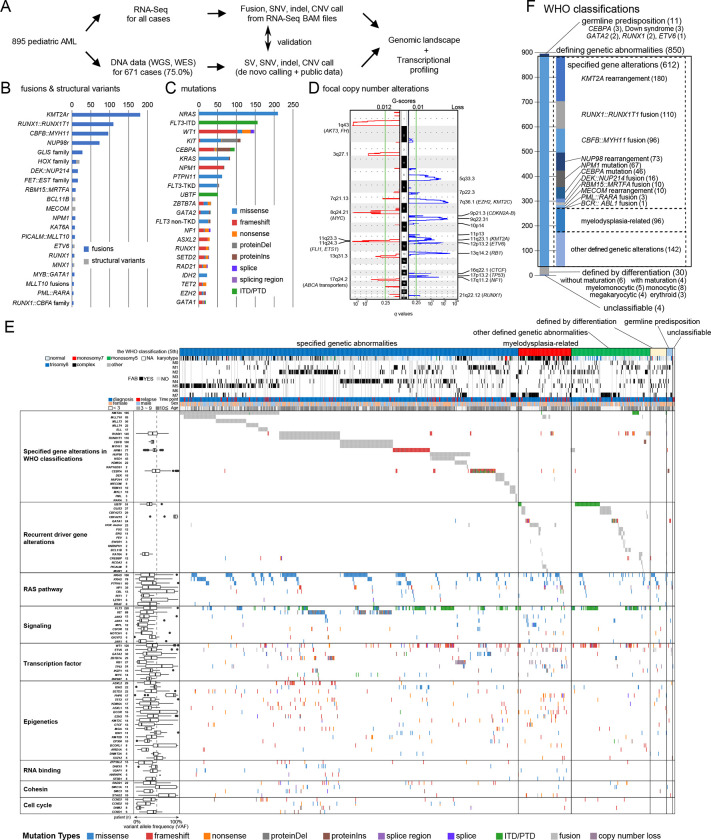
Comprehensive genetic characterization of pediatric acute myeloid leukemia (pAML) **A.** Study cohort of pediatric AML (n=895) and study design **B.** Recurrent pathogenic or likely pathogenic in-frame fusions (blue) and structural variants (SV: gray) detected in the entire cohort. Fusions included only in-frame fusions, and SVs included out-of-frame fusions resulting loss of the C-terminus of the protein and alterations detected from WGS data using CREST. **C.** Recurrent pathogenic or likely pathogenic somatic mutations. Colors represent types of mutations. Bars in Fig.1B–C represent the total number of alterations in the cohort. **D.** Results of GISTIC analysis for focal chromosomal events (shorter than 90% of the chromosome arm). The left panel shows the enrichment of focal gains, and the right panel shows the enrichment of focal loses. Green lines show a significance threshold for *q* values (0.25). Representative genes in enriched regions are highlighted. **E.** The genomic landscape and the WHO classification of pAML. Representative genes from GRIN analysis or defining alterations are shown. **F.** Summary of the WHO classification of the entire cohort.

Pediatric AML samples were collected from previously published studies^5,9,10,17–26^ or clinical trials at St. Jude Children’s Research Hospital, resulting in a cohort of 895 unique pAMLs either at diagnosis (n=786, 87.8%) or at relapse (n=109, 12.2%) (Fig.1A and Fig.S1A, Table.S1). This pAML cohort showed a wide age distribution at diagnosis (range: 0–23.5, median 9.3), with peaks in infancy and adolescence (Fig.S1B). We first assessed the genetic landscape of these AMLs using RNA sequencing (RNA-Seq) data to detect fusions, internal or partial tandem duplications (ITD/PTD), copy number variants (CNV), as well as single nucleotide variants (SNV) and insertions and deletions (Indel) (Fig.1A–E, Table S2–9). For 671 cases (75.0%) with either whole genome sequencing (WGS, 59.2%) or whole exome sequencing (WES, 43.7%), we also collected processed data from publications or performed de novo calling for new cases included in this study (Fig.1A and Fig.S1C).

Pathogenic fusions or structural variants (SV) were identified in 627 patients (70.1%). Most of these are recurrent and class-defining in pAML (e.g., *KMT2A*r: 20.2%, *RUNX1::RUNX1T1*: 12.3%, Fig.1B, Fig.1E, Table.S6), whereas we also found fusions recurrent in other leukemias, such as *SET::NUP214*^27^ (n=1) or *SFPQ::ZFP36L2*^28^ (n=1). Mutational profiling revealed 1,947 pathogenic or likely pathogenic somatic mutations in 757 (84.6%) patients, including class-defining *NPM1* (68 patients: 7.6%) and *CEBPA* (49 patients: 5.5%) mutations (Fig.1C, Fig.1E, Table.S7–8). The majority of mutations were in genes involved in signaling pathways (n=874), epigenetics (n=313), and transcription factors (n=440). RAS pathway mutations were most frequent, with 37.5% (336/895) having at least one RAS-related mutation and 21.1% of those (71/336) having mutations in multiple RAS pathway genes. Among CNVs, we frequently observed gains of chromosome 8 (7.2%) or chromosome 21 (6.5%) and loss of the long arm of chromosome 5 (5q-: 1.5%) or chromosome 7 (3.9%) (Fig. 1D, Fig.S1E Table.S9). Enrichment of focal deletions involving *RB1*^26^ (13q14: 2.9%), *ETV6*^29^ (12p13: 2.1%), *NF1*^30^ (17q11: 2.0%), and *TP53* (17p13: 2.0%) were also observed in this cohort. In addition, underappreciated focal gains involving *AKT3*^31^ and *FH*^32^ (1q43: 3.0%) or *ABCA* transporters (17q24: 2.3%) were identified, suggesting their possible importance in leukemogenesis. GRIN (genomic random interval) analysis^33^ identified 143 genes significantly altered in the entire cohort (Fig.1E, Fig.S2A-B, Table.S10). Consistent with previous reports, RAS-related mutations or *FLT3*-ITD with variable variant allele frequencies (VAFs) were highly co-occurring with class-defining alterations (Fig.1E, Fig.S2). In contrast, mutations in *UBTF* or *CBFB* genes were predominantly found in cases without a defining driver alteration, as previously shown^10,34^, suggesting that these alterations define subgroups with distinct molecular characteristics.

Based on these collective data, we classified pAMLs using current WHO and ICC systems (Fig.1E–F, Fig.S1E-F, Table.S1), and the frequencies of major classifications are consistent with cytogenetic profiles of European pediatric AML cohorts^35,36^. In our pAML cohort, 68.3% of cases had specified genetic alterations in WHO^5th^, 10.7% of cases were defined as “acute myeloid leukemia, myelodysplasia-related” (AML-MR), and the remaining cases with rare fusions or no defining alteration were classified as “acute myeloid leukemia with other defined genetic alterations” (15.9%) or by differentiation stages (3.4%). In contrast, 95.0% of adult AMLs can be classified either by specific gene alteration (67.1%) or as AML-MR (27.8%)^37^, emphasizing the need for a more comprehensive classification of pAML based on the unique biology of childhood AML.

### Molecular categories of pAML defined by mutually exclusive gene alterations

**Figure 2:**
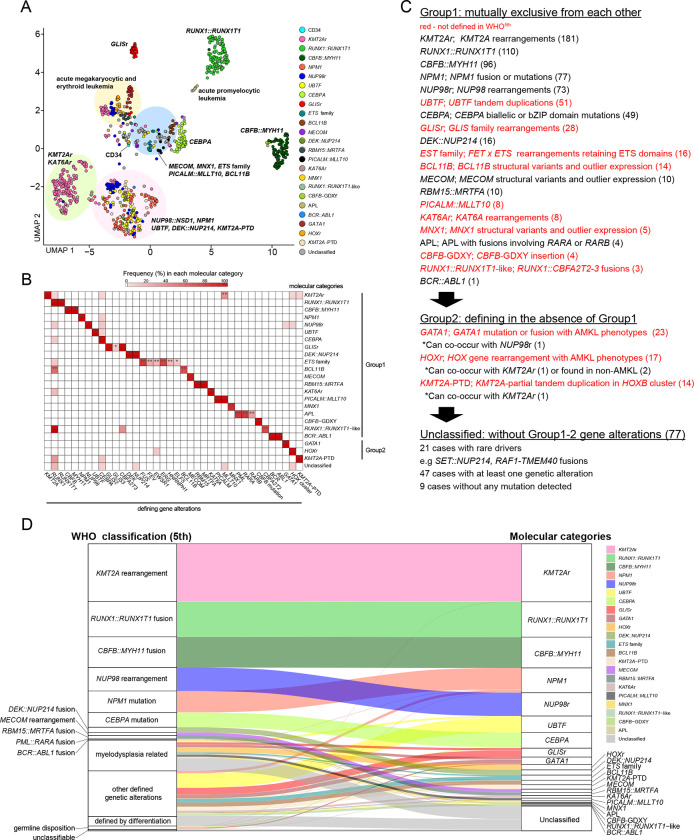
Molecular categories defined by mutually exclusive gene alterations **A.** UMAP plot of the entire pAML cohort (n=895) and cord blood CD34+ cells (normal controls: n=5) using top 320 variable genes. The colors of each dot denote the molecular categories of the samples. Representative category names are shown, and large clusters are highlighted in circles. **B.** A heatmap showing frequencies of defining gene alterations represented by the color. Statistical significance was assessed by two-sided Fisher’s exact test to calculate p values of co-occurrence, followed by the Benjamini-Hochberg adjustment for multiple testing to calculate *q* values (*P<0.05, **q<0.05). **C.** Definition of molecular categories and diagnostic flow. Molecular categories not defined in WHO^5th^ are highlighted in red. **D.** A ribbon plot showing the association between WHO classification and molecular categories. Colors represent molecular categories of samples

We and others have shown that class-defining driver alterations are associated with specific expression patterns^10,38,39^ or that allele-specific and outlier expression of *MECOM*^40,41^, *BCL11B*^42^, *or MNX1*^43^ by SVs can define disease subtypes. We then integrated the mutational landscape with expression profiling to define granular molecular categories for pAML (Table.S11). UMAP analysis^44,45^ of transcriptional data revealed tight clustering of classes defined in WHO^5th^, including *KMT2A* rearrangement (*KMT2A*r), *NUP98* rearrangement (*NUP98*r), *RUNX1::RUNX1T1, CBFB::MYH11, NPM1* mutation, *CEBPA* mutation, and *DEK::NUP214*, suggesting subtype-specific expression patterns (Fig.2A, Fig.S3A-B).

We noted that the clustering is also driven in part by differentiation status represented by marker gene expression, FAB (French-American-British) classification, or cellular hierarchy^46^ (Fig.S3C-E), contributing to heterogeneity within large categories such as *KMT2A*r or *NUP98*r (Fig.2A, Fig.S3A, S4A). Diffusion maps^47^ confirmed similar patterns of clustering and differentiation status (Fig.S3A-E). Cases with *NPM1* fusions or indels outside the N-terminus^48^ clustered with canonical *NPM1* mutations, and thus we assigned them to the *NPM1* category (Fig.S4A), and similarly, *TBL1XR1::RARB*^49^ to the APL (acute promyelocytic leukemia) category. For the *MECOM* category, we noted a case showing outlier and allele-specific expression of *MECOM* without any evidence of rearrangements, leaving other possible dysregulation mechanisms, such as enhancer amplification^42^ (Table.S12). Among the remaining cases without class-defining alterations, we found that the following alterations were also mutually exclusive with each other and thus were defined as independent molecular categories: *UBTF* tandem duplications^10^, *GLIS* family (*GLIS2–3*) fusions^9^, fusions of *FET* and *ETS* family genes^50,51^ (e.g., *FUS::ERG*), *BCL11B* structural variants^42^, *PICALM::MLLT10^52^*, *KAT6A* rearrangements^53^, *MNX1* structural variants^54^, *RUNX1* fusion with *CBFA2T2–3*^55,56^ *(RUNX1::RUNX1T1*-like*)*, and newly reported *CBFB* insertions (*CBFB*-GDXY)^34^ (Fig.2A–C). *GATA1* fusions (e.g., *MYB::GATA1*) or mutations^57^, rearrangements involving *HOX* cluster genes^26^, and PTD of *KMT2A*^58^ could rarely co-occur with the above-mentioned category-defining alterations (Fig.2B). However, they are still predominantly found in cases without category-defining alterations, and cases were assigned to these categories only with consistent expression patterns and without previously explained driver alterations (Fig.2A–C). In contrast, defining mutations of AML-MR in WHO^5th^ were overall rare (range: 0.1–2.1%), frequently co-occurred with other defining alterations (e.g., *EZH2* in *PICALM::MLLT10*), and could be found in various clusters rather than as a distinct group (Fig.S3A, F), leading to its exclusion as a defining category for pAML. These 23 molecular categories with defining driver alterations and characteristic expression profiles covered 91.4% (818/895) of the pediatric AML cohort (Fig.2D, Table.S1).

### Clinical, mutational, and transcriptional characterization of the molecular categories

**Figure 3:**
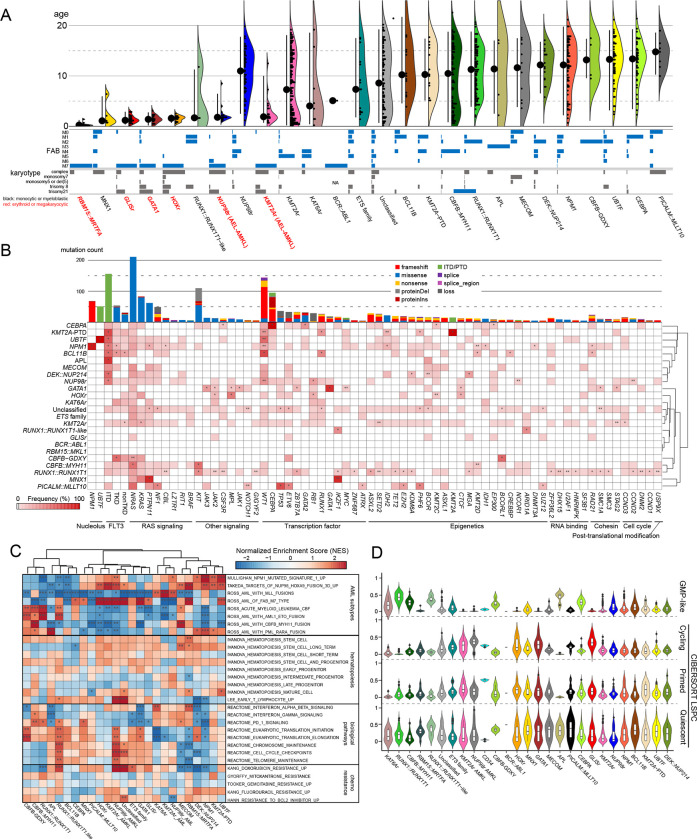
Clinical and molecular profiles of molecular categories **A.** Clinical background of molecular categories. **Upper row.** Violin plots showing age distribution within each molecular category. Large dots and bars represent the median and the range of 2.5~97.5 percentiles, respectively. Small dots represent individual patients’ ages. **Bottom row.** Frequency of FAB and karyotype in individual categories. **B.** Mutational heatmap showing mutation frequencies in each molecular category. The color of each panel represents the frequency of a mutation in each molecular category, and the statistical significance was assessed by two-sided Fisher’s exact test to calculate p values of co-occurrence followed by the Benjamini-Hochberg adjustment for multiple testing to calculate *q* values (*P<0.05, **q<0.05 after the adjustment). Bars on the top panel show the frequency of mutations in the entire cohort, and the colors represent mutation types. Molecular categories are clustered according to Ward clustering using the Euclidean distance of the frequency matrix. Genes are grouped according to the functional annotations. **C.** A heatmap showing normalized enrichment scores (NES) and false discovery rates (FDR) of gene set enrichment analysis (GSEA) of each molecular category. Colors denote NES, and asterisks show FDR (*FDR<0.05, **FDR<0.01, ***FDR<0.001) **D.** Violin plots showing cellular hierarchy scores in each molecular category inferred by CIBERSORT. Lines of the box represent 25% quantile, median, and 75% quantile. The upper whisker represents the higher value of maxima or 1.5 × interquartile range (IQR), and the lower whisker represents the lower value of minima or 1.5 × interquartile range (IQR). Dots show outliers. LSPC stands for leukemic stem and progenitor cells.

Establishing updated molecular categories for pAML allowed for the investigation of clinicopathological associations. Categories with acute megakaryoblastic or erythroid leukemia (AMKL/AEL) phenotypes are clearly enriched in infants, whereas CBF leukemias and mutation-defining leukemias (e.g., *UBTF*, *NPM1*, *CEBPA*) were enriched in adolescents and young adults (Fig.3A, Fig.S4A-B). Notably, among *KMT2A* fusion partners, *MLLT3* and *MLLT10* were found in both monocytic AML and AMKL; however, these fusions preferentially show AMKL phenotypes in infants (Fig.S4A), suggesting that AMKL phenotypes are defined both by types of driver alterations and by the developmental stages as discussed in *GATA1*-driven AMKL in Down syndrome patients^59^ or *GLIS2::CBFA2T3*-driven AMKL^60^. Overall, however, each molecular category showed variable morphological features represented by FAB classification except categories with APL (M3) or AMKL (M7) phenotypes (Fig.3A). Likewise, conventional cytogenetics also had a limited role. Complex karyotypes, which also define AML-MR^11^, were frequently observed in *MNX1*, *HOX*r, and *PICALM::MLLT10* categories. Additionally, as many of these category-defining alterations are cytogenetically cryptic (e.g., *NUP98*r^8^ or *GLIS* family^9,61^) or somatic mutations (e.g., *CEBPA*, *UBTF*, or *GATA1*), sequencing approaches are needed for the appropriate molecular diagnosis of pAML.

We next explored the association between initiating driver alterations and cooperating mutations, as some cooperating mutations co-occur and act synergistically with specific driver events^,5,62–64^. Signaling alterations were broadly found in 66.6% of patients, whereas each mutation showed distinct patterns among molecular categories with variable VAFs (Fig.1E, Fig.3B, Table.S7). Among *RAS* mutations, *NRAS* mutations were broadly found and enriched in *CBFB::MYH11*, whereas *KRAS* mutations were enriched in *KMT2A*r. Similarly, *FLT3*-ITD showed strong enrichment in *NUP98*r, *NPM1*, *UBTF*, *KMT2A*-PTD, *DEK::NUP214*, APL, and *BCL11B categories,* accounting for 76.3% of *FLT3*-ITD+ cases, whereas 74.0% of *FLT3*-TKD (tyrosine kinase domain) were found in *KMT2A*r, *NPM1*, and CBF-AMLs. Similarly, *WT1* mutations were specifically enriched in *NUP98*r, *UBTF*, and *BCL11B* and highly co-occurring with *FLT3*-ITD (Fig.3B, Fig.S2A).

We further evaluated gene expression signatures among molecular categories. Top variable genes across the cohort enriched genes in development (e.g., *HOX* genes), differentiation, or inflammation (Fig.S5A, Table.S13), consistent with previous reports that the heterogeneity of AML can be partly attributed to differentiation status^1,46,65^. Gene set enrichment analysis (GSEA) first confirmed that expression profiles of major categories were congruent with previous reports^38,66,67^ (Fig.3C, Table.S14). The new categories we propose in this study show similarities and differences with clearly defined categories. For example, *UBTF* showed expression signatures similar to *NPM1* and *DEK::NUP214*, while *KAT6Ar* was similar to *KMT2A*r, suggesting shared biological mechanisms. In addition, genes involved in signaling pathways, immunity, or drug resistance showed unique enrichment across categories (Fig.3C). Weighted gene co-expression network analysis (WGCNA)^68^ confirmed that each category showed characteristic patterns of active gene networks associated with specific biological functions (Fig.S5B, Table.S15).

Given recent adult AML-focused studies using expression profiling to uncover the associations of cellular stemness^69,70^ or hierarchy^46,71^ with prognosis or drug response, we investigated these features in our pAML dataset. We observed unique patterns of stemness and cellular hierarchy scores in each category. Molecular categories known to have a good prognosis (*RUNX1::RUNX1T1*^35,36^, *CBFB::MYH11*^35,36^, and *CEBPA^72^*) tended to have high GMP scores (median >0.20) (Fig.3D, Fig.S5C), with the exception of the low GMP scores (median: 0.047) and mid-high stemness-related scores in *NPM1*. Also, *KMT2A*r, associated with poor prognosis^35,73,74^, showed low stemness-related scores and variable differentiation-related scores. Various prognostic scores (e.g., LSC17^69^, iScore^65^) also correlated with molecular categories (Fig.S5D). These data collectively demonstrate that molecular categories are associated with unique pathophysiological characteristics, whose clinical association needs to be assessed based on detailed molecular categories.

### Superfamilies defined by *HOX* gene expression profiles

**Figure 4:**
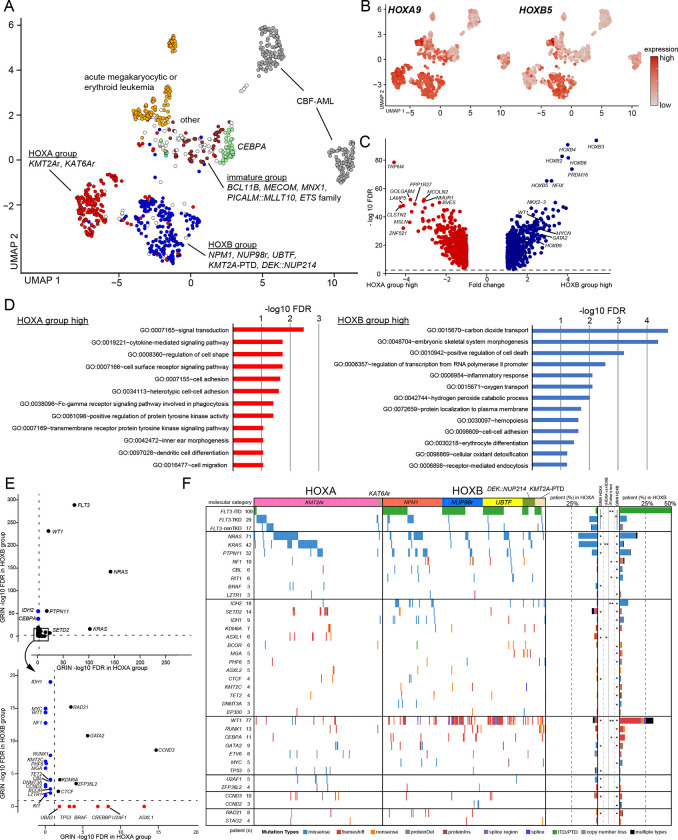
Categories demarcated by HOXA and HOXB cluster expression **A.** UMAP plot showing groups of molecular categories based on UMAP clustering and HOX cluster gene expression profiles. **B.** *HOXA9* and *HOXB5* expression on UMAP plot. The dot colors represent the relative expression of the genes. **C.** A volcano plot showing differentially expressed genes (DEG) between HOXA and HOXB groups. Genes with absolute fold change > 2 and FDR < 0.05 are considered DEGs. Representative gene names are shown. **D.** GO term analyses of genes with significantly high expression in each HOX group by DAVID. Bars represent logged FDR. **E.** Plots showing results of GRIN analyses in HOXA group (horizontal axis) and HOXB group (vertical axis). Genes with FDR<0.1 in either HOXA or HOXB groups are shown. Red or blue dots show genes enriched only in either HOXA or HOXB groups, respectively. The dotted lines represent thresholds for statistical significance (FDR<0.05). **F.** A mutational heatmap comparing patterns between HOXA and HOXB groups. Colors represent mutation types, and molecular categories are annotated on the top. Bar plots on the right show frequencies of mutations in HOXA and HOXB groups. Statistical significance of GRIN analysis in HOXA and HOXB groups (*FDR<0.05) and two-sided Fisher’s exact test between HOXA and HOXB groups (*P<0.05, **q<0.05 after the Benjamini-Hochberg adjustment) are also shown. GRIN results for *FLT3* are for the entire gene, while Fisher’s tests were performed separately for ITD, TKD, and non-TKD mutations.

These molecular categories also showed inter-categorical similarities, forming large clusters of AMKL/AEL, immature AML, CBF leukemias, *CEBPA*, and two clusters demarcated by *HOXA* and *HOXB* cluster gene expression (Fig.2A, Fig.4A–B). The cluster with high *HOXA* gene expression and low *HOXB* expression consisted mainly of *KMT2A*r and *KAT6A*r (herein referred to as HOXA categories), and the other cluster characterized by high expression of both *HOXA* and *HOXB* genes included *NPM1*, *NUP98*r, *UBTF*, *KMT2A*-PTD, and *DEK::NUP214* (HOXB group) (Fig.4A, Fig.S6A). Overall, HOXA and HOXB groups, not including those with AMKL features, account for 17.8% and 22.4% of the cohort, respectively. Differential gene expression analyses revealed that pAMLs in HOXB group had high expression of stemness-related genes (*PRDM16* and *NKX2–3*) or differentiation genes *(CD96* and *WT1*) (Fig.4C–D, Table.S16). In contrast, HOXA group cases showed high expression of monocyte or signaling-related genes. GRIN analysis also revealed striking differences in mutational patterns between HOXA and HOXB groups (Fig.4E–F, Table.S17). *FLT3* was significantly altered in both HOX groups but with different mutation types; *FLT3*-TKD was dominant in HOXA group, and *FLT3*-ITD was prevalent in HOXB group, accounting for 67.3% of *FLT3*-ITD+ patients (Fig.4F, Fig.S6B). *WT1* mutations were preferentially found in HOXB group (56.6%). *FLT3*-ITD^75^ and WT1 mutations^17,76^ have been associated with poor prognosis: however, these data suggest that these mutations highly confound with specific driver alterations that converge on a common expression signature. *KRAS* mutations were strongly associated with HOXA group and rare in HOXB group (22.2% and 4.2%, respectively). In comparison, *NRAS* mutations were prevalent in both HOXA and HOXB group (22.7% and 19.3%) (Fig.4F); however, among *NRAS* mutations, *NRAS p.*G12–13 mutations were comparable in both categories, while *NRAS p*.Q61 mutations were more frequent in HOXA group (Fig.4E, Table.S7). It is well-established that each RAS mutation has preferential distribution among cancer subtypes^77^_._ Expression levels and differences in the downstream signaling are postulated as the possible mechanisms, and similarly, between *FLT3*-ITD and TKD^78^, while at the RNA levels, these genes were homogenously expressed in this pAML cohort (Fig.S6B). These molecular category-dependent mutational patterns may reflect different signal dependencies, potentially offering less toxic targeted therapies guided by these biological insights.

Along with the global distinction between HOXA and HOXB groups, we also noted heterogeneity within each HOX cluster. The HOXA cluster consisted of subclusters characterized by *MECOM* or *LAMP5* expression (Fig.S7A-C, Table.S18), harboring most *KMT2A*r cases (120/181; 66.3%). Notably, the larger subcluster expressed *XAGE1* family genes specifically (Fig.S7B-C), which encode members of testis-specific proteins postulated as therapeutic targets in various tumor types^79^. Also, the remaining *KMT2A*r cases were clustered with other categories with *HOXB* expression or AMKL less frequently (Fig.S4A and S7A). These clustering patterns were associated with fusion partners (e.g., *KMT2A::ELL* in the HOXB cluster) or age (younger age with AMKL and outliers), but the associations were not exclusive (Fig.S7D-E). Among *KMT2A*r, fusion partners and *MECOM* expression have been reported to be prognostic^73,74^; however, our data suggest considerable heterogeneity in expression patterns not explained by only fusion partners or *MECOM* expression. The HOXB cluster showed similar heterogeneity represented by cellular hierarchies (Fig.S7F-G). These heterogeneities were occasionally associated with molecular categories or somatic mutations but were not exclusive (Fig.S7G-H), with possible factors, including cell-extrinsic factors^65,80^ to be further investigated.

### Molecular basis of AML without defining gene alterations

**Figure 5:**
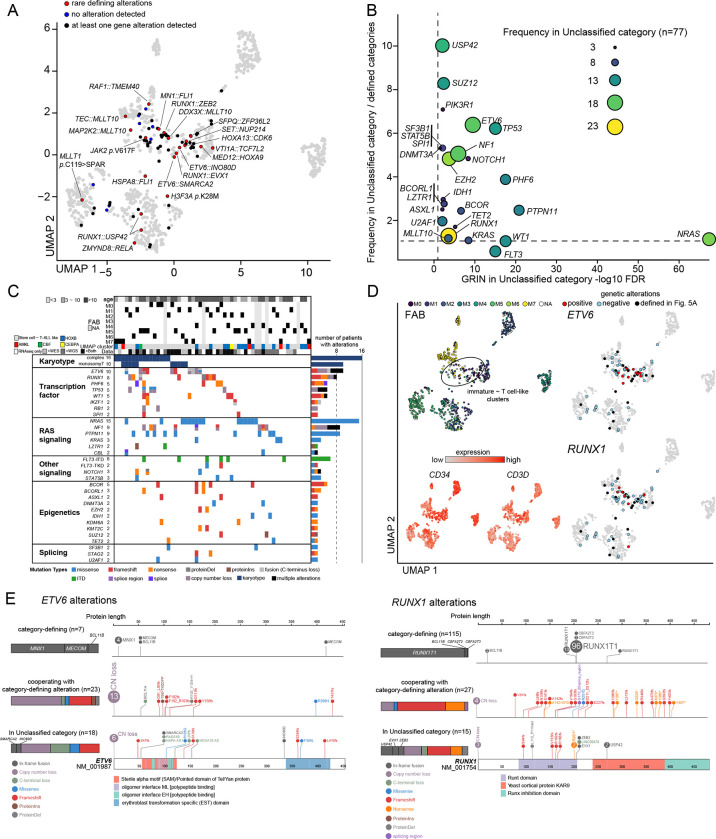
Characterization of cases without category-defining alterations **A.** UMAP plot showing cases without category-defining alterations. Red dots represent cases with rare recurrent gene alterations, blue dots represent cases for which no pathogenic alteration was found, and black dots represent cases with at least one gene alteration not defining the phenotype. **B.** A plot showing the FDR of GRIN analysis for the Unclassified category (horizontal axis) and relative enrichment of the alteration in the Unclassified category (vertical axis). The dot sizes and colors denote the Unclassified category’s frequency, which included fusions, mutations, copy number loss and gain, and copy-neutral heterozygosity. **C.** A mutational heatmap of the Unclassified cases, including complex karyotypes and monosomy 7. Patients’ age, FAB, and UMAP clustering are annotated on the top. Colors represent mutation types. **D.** UMAP plots showing FAB (**top-left**), *CD34* or *CD3D* expression (**bottom-left**), and cases with *ETV6* alterations (**top-right**) and *RUNX1* alteration (**bottom-right**). **E.** Patterns of alteration in *ETV6* (**left**) and *RUNX1* (**right**). Category-defining fusions are shown in the top row, alterations co-occurring with category-defining alterations in the middle row, and alterations in the Unclassified category in the bottom row. Bars represent a relative fraction of alteration in each group; the colors denote the alteration types.

Seventy-seven Unclassified cases remained after assignments into these 23 molecular categories. Twenty-one cases had recurrent driver alterations previously reported in the literature, whose sample sizes are insufficient to assess the biological characteristics (Fig.5A, Table.S19). These included rare or novel in-frame *RUNX1* fusions (n=2: *USP42*^81^, n=1: *EVX1*^82^ and *ZEB2*) and rare *MLLT10* fusions (n=1: *DDX3X*^83^, *TEC*^84^, and *MAP2K2*^26^), with the possibility of further categorization in a larger cohort. Also, in addition to hi-allelic burden *JAK2 p*.V617F mutation (n=1), we found candidate driver somatic mutations of *MLLT1 p*.C119>SPAR (n=1) and *H3F3A p*.K28M (n=1) in pAML with HOX gene expression (Fig.5A, Fig.S8A, Table.S7). These mutations resemble recurrent mutations in other pediatric cancer types with HOX gene expression and immature phenotypes (*MLLT1 p*.C118QPPG in Wilms tumor^85^ or *H3F3A p*.H28M in high-grade glioma^86^), postulating a shared mechanism of tumorigenesis among these pediatric neoplasms.

This series of genomic characterization did not find any pathogenic alterations in 9 cases of the remaining 56 Unclassified cases, partly attributed to the lack of WGS data for 8 of these cases. The rest had at least one pathogenic but not subtype-defining alteration enriched in *ETV6*, *RUNX1*, *TP53*, and RAS pathway genes (Fig.5B–C, Table.S19–20), in addition to complex karyotypes or monosomy 7 (Fig. 3A). Of note, complex karyotypes or *NRAS* mutations were found broadly in various clusters, whereas non-canonical *ETV6* and *RUNX1* alterations were found preferentially in clusters comprised of FAB M0–1 cases. These clusters are associated with immature or T cell-like signatures (Fig.5D, Fig.S8B, Table.S21), consistent with a new entity of AMTL^87^. Although various *ETV6* or *RUNX1* alterations can co-occur with other defining alterations or be class-defining (e.g., *RUNX1::RUNX1T1*), the mutations in the Unclassified category are commonly loss-of-function (Fig.5E). Given that germline mutations of *RUNX1* or *ETV6* are associated with leukemia with incomplete penetrance^88,89^, these data suggest somatic alterations of these genes also require additional mutations for leukemia development, which may cooperatively define the immature leukemic phenotypes in the absence of other defining alterations. Further accumulation of genomic data and experimental models will be necessary to understand the stepwise leukemogenesis and the clinicopathological features of immature pAML with these mutations.

### Clinical association of molecular categories

**Figure 6:**
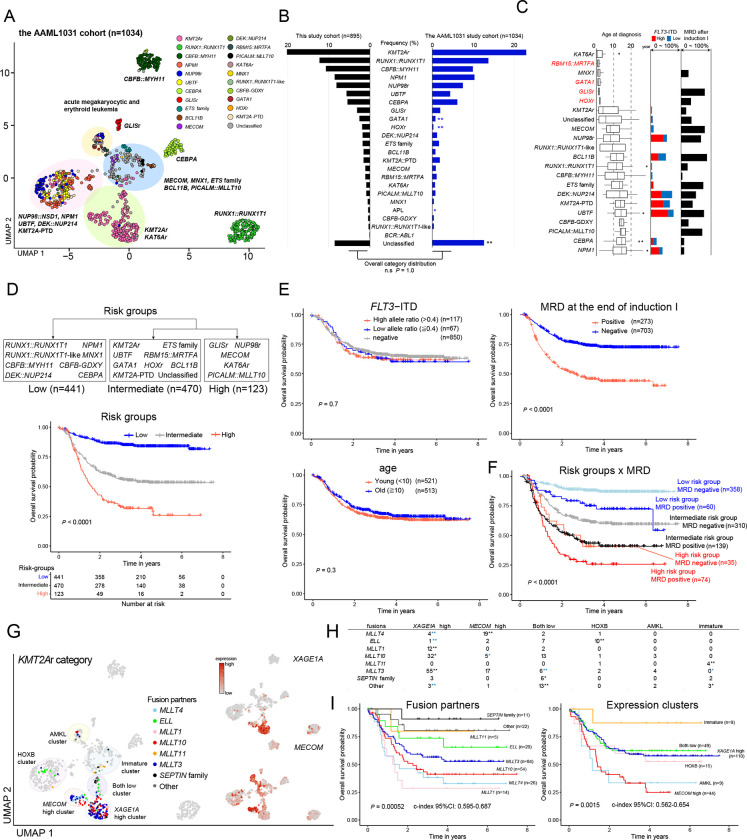
Clinical association of molecular categories **A.** UMAP plot of transcriptome data of the AAML1031 cohort (n=1,034) using top340 variable genes. The dot colors denote molecular categories assigned to the samples according to genomic profiling using the same pipeline as this study cohort. Representative category names are shown, and large clusters are highlighted in circles. **B.** Frequency of molecular categories in the AAML1031 cohort. Asterisks denote the statistical significance of the frequency of each category assessed by two-sided Fisher’s exact test followed by the Benjamini-Hochberg adjustment (*P<0.05, **q<0.05, blue: fewer and black: more in the AAML1031). **C.** Clinical features of molecular categories showing age at diagnosis (**left**), *FLT3*-ITD status (**mid**), and MRD (minimal residual disease) positivity at the end of induction (**right**). Molecular category names associated with megakaryocytic phenotypes are highlighted in red. Lines of the box represent 25% quantile, median, and 75% quantile. The upper whisker represents the higher value of maxima or 1.5 × interquartile range (IQR), and the lower whisker represents the lower value of minima or 1.5 × interquartile range (IQR). **D.** Grouping of molecular categories into Low, Intermediate, and High-risk groups by recursive partitioning (**top**) and Kaplan-Meier curves of overall survival of patients in each risk group (**bottom**). **E.** Kaplan-Meier curves and statistical significance of overall survival of patients with known prognostic factors (*FLT3*-ITD status: **top-left**, age: **bottom-left**, MRD positivity at the end of the induction I: **top-right**). **F.** Kaplan-Meier curves of overall survival of patients in six risk strata using risk groups (Low-Intermediate-High) and MRD positivity. **G.** Distribution of *KMT2A*r cases among transcriptional clusters on UMAP plot, colors representing fusion partners (**left**) and *XAGE1A* and *MECOM* expression, colors representing relative expression (**right**) on UMAP plot. **H.** The association of fusion partners of KMT2Ar among different clusters. **I.** Kaplan-Meier curves of overall survival of patients with each fusion (**left**) and in each cluster (**right**). For survival curves in **D**, **E**, **F**, and **I**, statistical significance was assessed by Cox Proportional-Hazards models, and *P* values are shown in the plot. For the validity of prediction by *KMT2A*r fusion partners and clusters in **I**, c-index scores assessed by bootstrapping were shown below the plots. For **I,** statistical significance of the enrichment and exclusivity were assessed by two-sided Fisher’s exact test followed by the Benjamini-Hochberg adjustment (*P<0.05, **q<0.05, blue: exclusive, black: enriched).

Although the association between *KMT2Ar* or *NUP98*r with poor outcomes is well-appreciated, the clinical associations of new molecular categories have been discussed only in separate studies^10,26^. To address this deficiency and translate them into a clinical framework, we investigated the outcomes of these molecular categories using the COG AAML1031 clinical study^15^ (n=1,034, Table.S22). Analyses of the AAML1031 RNA-Seq data using the same pipeline revealed similar clustering of molecular categories (Fig. 6A) and the overall category frequencies (Fig. 6B). The AAML1031 cohort confirmed the association of molecular categories with age and *FLT3*-ITD status (Fig.6C) and showed variable MRD (minimal residual disease) positivity among molecular categories. We first assessed the clinical association of the categories using recursive partitioning models^90^ for censored event time data, which revealed three groups with distinctive prognoses (Fig.6D, Fig.S9A-B). The grouping of major categories aligns with previous reports (e.g., *RUNX1::RUNX1T1* (n=141), *CBFB::MYH11* (n=102), and *CEBPA* (n=63) in low-risk^35,36,72^) except *DEK::NUP214* (n=17) in low-risk, historically regarded as high-risk^36,91^. We confirmed the known association of *GLIS*r^61^ (n=20), *MECOM*^92^ (n=11), *PICALM::MLLT10*^93^ (n=8), and *KAT6Ar*^93^ (n=7) with poor outcomes, while new categories of *MNX1* (n=4), *RUNX1::RUNX1T1*-like (n=4), and *CBFB*-GDXY (n=4) were assigned to low-risk. Univariate analyses of other risk factors revealed that age and *FLT3*-ITD were not prognostic, whereas MRD positivity and cellular hierarchy scores (GMP-like, cDC-like, and cycling LSPC) were associated with the overall survival (Fig.6E, Fig.S9C-D, Table.S23). A Cox proportional hazards model using risk groups and prognostic factors showed that hierarchy scores did not significantly contribute to prognosis (Fig.S9E), whereas risk groups and MRD positivity were independently prognostic (Table.S23). These data led us to establish a simple predictive framework solely based on molecular categories and MRD positivity, resulting in six risk strata with granular outcome prediction (Fig.6F and Fig.S9F-G), whose prognostic values were validated using the AML08 trial^14^ (n=211, Fig.S9H-J, Table.S24).

With these transcriptional and outcome data, we also investigated the clinical association of transcriptional heterogeneity within major molecular categories. Among *KMT2A*r, fusion partners or *MECOM* expression^73,74^ also confound in the AAML1031 cohort (Fig.6G–H). Cox hazard models showed that both fusion partners and expression clusters are prognostic (*P* =0.00052 and 0.0015, respectively), with fusions with *SEPTIN* family and *ELL* or immature expression patterns associated with favorable outcomes (Fig.6I). Bootstrapping showed that the association of fusion partners or expression clusters with prognosis did not significantly differ (difference in C-index of 95% bootstrap interval for fusions and expression clusters: −0.025–0.093). Although HOXB categories of *NUP98*r, *NPM1*, and *UBTF* also showed heterogeneity of expression patterns, their outcomes were not associated with UMAP clusters or *FLT3*-ITD status (Fig.S10), and further mutational profiling outside the clinical standard (e.g., *WT1* mutations) may be required to risk-stratify within these categories further.

## Discussion

In addition to known enrichment of chromosomal events like t(11,x) in pediatric patients with AML, advances in sequencing technology have identified additional pediatric-specific driver alterations^9,10,34^. This prompted us to comprehensively investigate the increasingly complex genomic landscape of pAML in the context of the latest classification systems for hematological malignancies (WHO^5th,11^ and ICC^12^) and to develop a pAML-focused categorization schema based on the unique disease biology of childhood AML. In this study, we systematically categorized our pAML cohort of 895 patients using an RNA-Seq-based approach, resulting in 23 molecular categories defined by mutually-exclusive driver alterations, covering 91.4% of the entire cohort. Of these 23 categories, 12 are not currently defined by WHO^5th^. This includes common categories like *UBTF*, *GLIS*r and *GATA1*, which were otherwise categorized as “acute myeloid leukemia, myelodysplasia-related” or “acute myeloid leukemia, other defined gene alteration” in the current WHO classification. Notably, myelodysplasia-related (MR) mutations or chromosomal alterations often co-occur with many pAML category-defining alterations and override them in WHO^5th^ despite these MR alterations not driving consistent patterns of gene expression. Considering that the current classification systems are mainly based on evidence from adult AML^94,95^ and pediatric myelodysplasia syndrome (MDS) is rare^22^, we propose an alternative framework for pAML to better reflect the unique genetic and clinical landscape.

These molecular categories show unique expression and mutational profiles, whereas some categories also show critical similarities, which can suggest common molecular mechanisms and potentially therapeutic options. In particular, we noticed two large clusters characterized by unique HOXA-B expression profiles. Molecular categories with HOXB signatures were strongly associated with *FLT3*-ITD and *WT1* mutations, whereas those with HOXA signatures were associated with *RAS* mutations. Considering that AMLs with *KMT2Ar*, *NUP98*r, and *NPM1* have been shown to be dependent on KMT2A/Menin^96–98^, and that a Menin inhibitor (SNDX-5613) targeting *KMT2A*r and *NPM1* AML is in a clinical trial^99^ (NCT04065399), our data suggest that other subtypes marked by HOX expression, such as *UBTF* or *DEK::NUP214*, may also be candidates for Menin inhibitors. Thus, this approach may cover nearly 50% of pediatric AML. Also, the high frequency of *FLT3*-ITD in categories with HOXB expression implies that FLT3 signaling is closely related to biology and that a data-driven implementation of FLT3 inhibitors to HOXB subtypes can be effective.

Some cases without category-defining alterations could be characterized by rare fusion or mutations, which need further evidence to establish as a disease entity, including *MLLT1* and *H3F3A* mutations that are frequent and class-defining in Wilms tumor^85^ and glioma^86^, respectively. Considering that AML and Ewing sarcoma also share *ETS* family fusions^51^ (e.g., *EWSR1::ERG*), it would be intriguing to incorporate knowledge of these solid tumors to understand the biology behind pAML with these rare alterations. Also, enrichment of *RUNX1* or *ETV6* loss of function alterations in immature AML implies that these can be class-defining in the absence of other defining alterations and likely with specific cooperating mutations. These findings further suggest a continuum with other immature leukemias, such as early T-cell precursor (ETP)-ALL and mixed phenotype acute leukemias (T/My) which have similar mutational features^82,100^.

We further investigated the clinical outcomes of these molecular categories using two independent cohorts --the COG AAML1031 study and the St. Jude AML08 study. Using both cohorts, we show a strong association of new molecular categories with outcomes (e.g., *PICALM::MLLT10* and *KAT6A*r as high-risk, *CBFB*-GDXY as low-risk). These analyses also revealed confounding variables like molecular categories and known prognostic factors like *FLT3*-ITD status or cellular hierarchy scores. With this comprehensive profiling recognizing new pAML subtypes, we established a simple risk stratification using molecular categories and MRD. This strategy, however, heavily relies on the analysis of next-generation sequencing data. While the WHO classification requires targeted sequencing or WGS, we propose a diagnostic pipeline utilizing RNA-Seq which is highly sensitive for canonical and cryptic fusion calling, allows for categorization based on gene expression signatures, including outlier and allele-specific expression (*MECOM*, *BCL11B*, and *MNX1*), and provides limited but sufficiently sensitive mutation calling to enable our comprehensive molecular categorization strategy to newly diagnosed pAML.This approach is favored over current commercial panels commonly used for pAML, which either lack coverage of all the defining genes (e.g., *UBTF*) or are not suitable to detect complex structural variations that drive aberrant expression of *MECOM* or *BCL11B*. Given that clinical sequencing is not readily available globally and these molecular analyses require substantial expertise, robust and easy pipelines are needed for future and broad application of this framework for pAML in the general clinical setting.

## Online Methods

### Subject cohorts and sample details.

Tumor samples from patients with AML from the St. Jude Children’s Research Hospital tissue resource core facility were obtained with written informed consent using a protocol approved by the St. Jude Children’s Research Hospital institutional review board (IRB). Studies were conducted in accordance with the International Ethical Guidelines for Biomedical Research Involving Human Subjects. Samples for RNA sequencing (RNA-Seq: n=221), whole genome sequencing (WGS: n=54), and whole exome sequencing (WES: n=6) are newly sequenced in this study, and the rest of the data were obtained from previous publications^5,9,10,17–26^ or public databases (see details in Data availability and Table S1). For samples with multiple available data points, we included one representative time point with a high tumor purity and good RNA-Seq data quality. Cases were assigned to current WHO and ICC by board-certified hematopathologists (PK and JMK).

### Sample processing, library preparation, and sequencing.

For newly sequenced samples with low tumor purity (below 60%), the leukemic cell population was enriched either by flow cytometric sorting or T cell depletion by magnetic beads (EasySep Human CD3 Positive Selection Kit II, 17851, StemCell Technologies). For flow cytometric sorting, CD45^dim^CD33^dim^ positive population was sorted using anti-CD45 PerCP-Cyanine5.5 (eBioscience cat# 8045–9459-120) and anti-CD33 APC (eBioscience cat# 17–0338-42). CD34 gating using anti-CD34 PE (Beckman cat# IM1459U) was added depending on the positivity of each patient sample. Enrichment of the tumor population was confirmed flow cytometric analysis of the post-sorting samples (generally > 90%). Libraries were constructed using the TruSeq Stranded Total RNA Kit, with Ribozero Gold (20020598, Illumina) for RNA-Seq, the TruSeq DNA PCR-Free Library Prep Kit (20015963, Illumina) for WGS, and the TruSeq Exome Kit v1 (20020614, Illumina) for WES according to the manufacturer’s instructions. After library quality and quantity assessment, samples were sequenced on HiSeq2000 or 2500 (Illumina, RRID:SCR_020132, RRID:SCR_016383) instruments with paired-end (2 × 101 bp, 2 × 126 bp, or 2 × 151 bp) sequencing using TruSeq SBS Kit v3-HS (FC-401–3001, Illumina) or TruSeq Rapid SBS Kit (FC-402–4023, Illumina).

### RNA-Seq mapping, fusion detection, and large-scale copy number variant calling.

RNA reads from newly sequenced samples and from publications were mapped to the GRCh37/hg19 human genome assembly using the StrongARM pipeline^101^. Chimeric fusion detection was carried out using CICERO^102^ (v0.3.0). For the cases with only RNA-Seq data, RNAseqCNV^103^ (v1.2.1) was used to call large-scale copy number variants (CNV).

### Somatic mutation calling from RNA-Seq.

We initially performed RNA-based variant calling methods for detecting Single-nucleotide variants (SNV) and Insertions and deletions (Indel). Specifically, we applied the following approach to simultaneously account for germline polymorphisms (without germline control) and sequencing artifacts specific to RNA-Seq on a panel of 86 predefined genes previously reported to be significantly mutated in pediatric AML^5^ and myelodysplastic syndrome (MDS; Table.S5). Briefly, candidate SNVs/Indels were called by Bambino^104^ (v1.07) or RNAindel^105,106^ (v3.0.4), annotated by VEP^107^ (v95), and in turn, classified for putative pathogenicity with PeCanPie/MedalCeremony^108^. Candidate variants with putative pathogenicity were considered germline or artifacts if present in >5% of the cases. Candidate variants were further filtered if the number of supporting reads was ≤5 or if the variant allele fraction (VAF) was ≤5%. *UBTF* tandem duplications were detected by soft-clipped read counting in addition to CICERO and RNAindel as we previously described^10^.

### Whole genome and whole exome sequencing data analysis.

The previous genomic lesion calls for the cases (WGS; n=394, WES; n=284) from published studies^5,9,10,17,19–21,24,26^ were collected from their respective publications. For the unpublished cases with DNA data (WGS; n=136, WES; n=107), DNA reads were mapped using BWA^109,110^(WGS: v0.7.15-r1140 and v0.5.9-r26-dev; WES: v0.5.9-r26-dev and v0.5.9, RRID:SCR_010910) to the GRCh37/hg19 human genome assembly. Aligned files were merged, sorted, and de-duplicated using Picard tools 1.65 (broadinstitute.github.io/picard/). SNVs and Indels were called using Bambino^104^. Candidate SNVs and Indels were similarly classified for putative pathogenicity with PeCanPie/MedalCeremony as in somatic mutation calling from RNA-Seq. The counting of somatic mutations included all the pathogenic or likely pathogenic mutations detected by WGS, whereas mutation detection from cases with only RNA-Seq data is limited to the 86 preselected genes. Structural variations (SV) were analyzed using CREST^111^ (v1.0), and CNVs were analyzed using CONSERTING^112^ on the WGS data. CNVs were also called on cases with only WES DNA data using the following methods. Briefly, Samtools^113^ mpileup command was used to generate a mpileup file from matched germline and tumor BAM files with duplicates removed. If a matched germline was not available, a high-quality normal sample was used to pair with the tumor sample. VarScan^114^ (v2.3.5) was then used to take the mpileup file to call somatic CNVs after adjusting for normal/tumor sample read coverage depth and GC content. Circular Binary Segmentation algorithm^115^ implemented in the DNAcopy R package (v1.52.0) was used to identify the candidate CNVs for each sample. B-allele frequency info was also used to assess allelic imbalance.

### GRIN analysis for significantly mutated genes.

For the 895 AML cases, the genomic random interval (GRIN; v2.0) model^33^ was used to evaluate the statistical significance of the number of subjects with each type of lesion: fusions, CNVs (amplifications and deletions), copy neutral loss of heterozygosity (CN-LOH), SNV/indels, and tandem duplications in each gene. For each type of lesion, robust false discovery estimates were computed from *P* values using Storey’s *q* value^116^ with the Pounds-Cheng estimator of the proportion of hypothesis tests with a true null hypothesis^117^. FDR cutoff of <0.05 for the number of subjects with any one type of lesion overlapping the gene locus was used to obtain significantly mutated genes, where we focused on protein-coding genes and genes that are known or likely to be pathogenic in leukemia. We also excluded genes that are part of a large chromosomal gain, loss, or CN-LOH but not the target of the CNVs based on the GISTIC (Genomic Identification of Significant Targets in Cancer) analysis. Subgroup GRIN analyses for HOXA categories (n=167), HOXB categories (n=211) categories and the Unclassified category (n=77) were also performed using the same methods.

### GISTIC analysis for significant recurring copy-number alterations.

We used GISTIC (v2.0.23, RRID:SCR_000151) ^118,119^ to identify genomic regions that are significantly amplified or deleted across our 895 samples. Each aberration was assigned a G-score that considered the amplitude of the aberration as well as the frequency of its occurrence across samples. False discovery rate q values were then calculated for the aberrant regions, and regions with q values ≤0.25 were considered significant. A “peak region” was identified for each significant region with the greatest amplitude and frequency of alteration. In addition, a “wide peak” was determined using a leave-one-out algorithm to allow for errors in the boundaries in a single sample. The “wide peak” boundaries were more robust for identifying the most likely gene targets in the region. Each significantly aberrant region was also tested to determine whether it resulted primarily from broad or focal events (A broad event was set as >90% of the chromosome arm, whereas a focal event was ≤90%).

### Allele-specific expression estimation for *MNX1*, *BCL11B*, and *MECOM* categories.

For cases with both WGS and RNA-Seq available, SNP (single-nucleotide polymorphism) markers in the respective gene locus with ≥10x coverage that are heterozygous (defined as 0.2≤VAF≤0.8) in WGS and also present in RNA-Seq were extracted and a two-sided binomial test (with probability of success *P*=0.5) was performed on each marker for allelic imbalance in RNA expression. The median of binomial *P* values was used to assess allele-specific expression (ASE). For RNA-Seq only cases, SNP markers in the respective gene locus with ≥10x coverage and allelic imbalance (VAF≤0.2 or VAF≥0.8) support ASE.

### Germline variant curation methods.

We focused on 15 candidate genes relevant to AML that define specific categories in WHO^5th^ (Table.S25) and scanned for germline mutations in the cases with WGS or WES germline BAM files available (WGS n=367; WES n=354). For cases with germline mutation called in previously published studies^10,22^, we collected calls from the studies. For the remaining cases, the putative germline variants were called using Bambino^104^, annotated by VEP^107^, and in turn, classified for putative pathogenicity with PeCanPie/MedalCeremony^108^. We then used the following criteria to obtain the candidate germline variants: gnomAD (v2.1.1, RRID: SCR_014964)^120^ population allele frequency ≤0.001; read coverage SNV≥20 and Indel≥15; for SNV, variant allele frequency between 0.2 and 0.8; for Indel, ≥3 reads supporting the alternative allele. All candidate germline variants were comprehensively reviewed and classified as pathogenic, likely pathogenic, of uncertain significance, likely benign, or benign based on recommendations from the American College of Medical Genetics and Genomics and the Association for Molecular Pathology^121^ and the Clinical Genome Resource^122–125^ by a variant scientist (JLM).

### Gene expression data summarization, batch correction, dimension reduction, and clustering.

Reads from aligned RNA-Seq BAM files were assigned to genes and counted using HTSeq^126^ (v0.11.2, RRID: SCR_005514) with the GENCODE (RRID: SCR_014966) human release 19 gene annotation. The gene count matrix was generated, and for a gene to be considered as expressed, we required that at least 5 samples should have ≥10 read counts per million reads sequenced. The count data were transformed to log2-counts per million (log2CPM) using Voom^127^ available from R package Limma^128^(v3.50.3, RRID: SCR_010943). We corrected for library strand (stranded total RNA vs. unstranded mRNA) and batch effect between St. Jude and TARGET cases using the ComBat method available from R package SVA^129^(v3.42.0, RRID:SCR_012836). The R package Seurat^130–133^(v4.1.0, RRID:SCR_016341) was used for dimension reduction and sample clustering. Briefly, the top variable genes were selected using the “vst” method. The expression data were then scaled, and PCA (Principal Component Analysis) was performed on the scaled data using the top 320 variable genes. Dimension reduction was performed using UMAP^44,45^ (Uniform Manifold Approximation and Projection, RRID:SCR_018217) with the top 100 principal components, n_neighbors =15 and min_dist =0.2. Samples were clustered using the top 100 principal components by first constructing a K nearest-neighbor graph and then iteratively optimizing the modularity using Louvain algorithm with resolution=3.5. Dimension reduction was also performed by Diffusion maps ^47,134^ algorithm available in the R package destiny^135^ (v 3.10.0) using the same 320 genes with the default setting except for number of principal components n_pcs=50.

Differential gene expression analysis was performed by Limma^128^, and we set Log2 CPM = 0 if it is < 0 based on the Log2 CPM data distribution. *P* values were adjusted by the Benjamini-Hochberg method to calculate the false discovery rate (FDR) using R function p.adjust. Genes with absolute fold change > 2 and FDR < 0.05 were regarded as significantly differentially expressed. Gene Set Enrichment Analysis (GSEA)^136^ was performed by GSEA (v4.2.3, RRID: SCR_003199) using MSigDB gene sets c2.all (v7.5.1), comparing each category with the rest of the categories. Permutations were done 1000 times among gene sets with sizes between 15 and 1500 genes. Normalized enrichment scores (NES) and FDR for arbitrary gene sets representing hematopoiesis, leukemia phenotype, biological processes, and drug responses were extracted from the entire results and shown in a heatmap. Weighted correlation network analysis (WGCNA) was done by R package WGCNA^68^ (v1.70–3, RRID:SCR_003302) using top 2000 variable genes and default setting with the exception of block-wide module calculation with reassignThreshold = 0 and mergeCutHeight = 0.25. Functional annotation of top 320 variable genes, differentially expressed genes, and genes in WGCNA modules were performed with DAVID^137^ (v6.8), and results for GO term, biological process (GOTERM_BP_DIRECT) were exported. Inference of cellular hierarchy by CIBERSORT^138^ (RRID:SCR_016955) was performed by the web interface of CIBERSORTx in absolute mode with S-mode batch correction without a permutation as previously reported^46^. TPM values were used as input data, and Malignant Signature Matrix and Malignant Single Cell Reference Samples were used as previously described^46^, and the malignant cell populations were normalized to 1 to calculate the relative fraction scores, which were shown in UMAP space or violin plots. Prognostic scores of LSC17^69^, pLSC6^70^, ADE-RS^139^, and iScore^65^ were calculated as reportedly. Hierarchical clustering (RRID:SCR_014673) of expression data, mutual-exclusivity matrix, and GSEA scores were performed using the Euclidian distance and Ward method.

### Statistical test.

For discrete values of the molecular category and the mutation frequency in cohorts, statistical significance and mutual exclusivity were assessed by two-sided Fisher’s exact test and Pearson’s correlation. Adjustment of multiple testing was performed by the Benjamini-Hochberg method using p.adjust function on R when appropriate. For survival data, decision trees were established by a recursive partitioning method using R library rpart^90^ (v4.1.19, RRID:SCR_021777). Kaplan–Meier curves for the probability of overall survival (OS) and event-free survival (EFS) were constructed using R package survival (v3.3–1, RRID:SCR_021137). Events in the probability of EFS calculations were defined as relapse, death in remission by any cause, and non-response, which was included as an event at the date of diagnosis. The Cox proportional hazards model was used to calculate the statistical significance of individual prognostic factors by univariate analyses first, and significant factors were included in a multivariate analysis. Clinical association of the molecular categories were first assessed using the AAML1031 study (n=1034), and the results were validated using the AML08 cohort (n=211, independent from the AAML1031, a part of this study cohort). R statistical environment (R v4.0.2, RRID:SCR_001905) was used for statistical tests.

### Visualization.

Mutational heatmaps and mutations on individual genes were visualized using ProteinPaint (https://proteinpaint.stjude.org/). Heatmaps of expression data, mutual-exclusivity matrix, and GSEA scores were created by pheatmap function of R library pheatmap (v1.0.12, RRID:SCR_016418). Other data visualizations were performed by ggplot function of R library ggplot2 (v3.3.6, RRID:SCR_014601), survminer (v0.4.9), and base plot function in R statistical environment. Figures are incorporated and edited using Adobe Illustrator (2021, RRID:SCR_010279). Annotation of genes in mutational heatmaps depend on common knowledges, and the definition of RAS pathway genes included causative genes of Noonan or Noonan-like syndrome^140^ (*NRAS, KRAS, PTPN11, NF1, CBL, LZTR1, RIT1, BRAF, SOS1*, and *HRAS*).

### Data availability.

The genomic data and expression data newly generated in this study (RNA-Seq: n=221, WGS: n=54, WES: n=6) have been deposited in the European Genome-Phenome Archive (EGA, RRID:SCR_004944), which is hosted by the European Bioinformatics Institute (EBI), under accession EGAS00001005760. For the remaining RNA-Seq data for 588 cases, 401 are St. Jude cases, of which 274 cases with data from the publications are available either on EGA or St. Jude Cloud^9,10,18,20–24,26^ or on the original publication^25^. For the other 127 published cases^19^, we downloaded the BAM files from EGA (EGAS00001004701). Unpublished data (n=86) are also available on St. Jude Cloud under the PCGP study (https://permalinks.stjude.cloud/permalinks/PCGP, n=8) and the RTCG study (https://platform.stjude.cloud/data/cohorts?dataset_accession=SJC-DS-1007, n=78). Of the remaining WGS data for 394 cases, 207 are St. Jude cases, of which 115 cases with data from the original publications^9,10,20,21,24,26^ are available on either EGA or St. Jude Cloud, and for the other 92 published cases^19^, we downloaded the BAM files from EGA (EGAS00001004701). Unpublished WGS data (n=82) are also available on St. Jude Cloud under the RTCG study. For the remaining WES data for 314 cases, 275 are St. Jude cases, of which 155 with data from the original publications^9,10,18,20–24,26^ are available either on St. Jude Cloud or EGA, and for the other 120 published cases^19^, we downloaded the BAM files from EGA (EGAS00001004701). Unpublished WES data (n=101) are also available on St. Jude Cloud under the PCGP study (n=2) and the RTCG study (n=99).

The data generated by the TARGET initiative^5,17^ (n=187) is also available under accession phs000218 (TARGET-AML) and phs000465 (TARGET sub-study, data is available as a part of phs000218), managed by the NCI. Information about TARGET can be found at http://ocg.cancer.gov/programs/target. Other data generated in this study are available in the Supplemental tables or upon request to the corresponding author.
